# Metformin reverts aortic calcifications and elastin loss induced by an experimental metabolic syndrome

**DOI:** 10.1530/EC-24-0714

**Published:** 2025-01-17

**Authors:** Lucas Streckwall, Nancy Martini, Claudia Sedlinsky, León Schurman, María Virginia Gangoiti, Antonio Desmond McCarthy

**Affiliations:** Laboratorio de Investigación en Osteopatías y Metabolismo Mineral (LIOMM), Facultad de Ciencias Exactas, Universidad Nacional de La Plata, La Plata, Argentina

**Keywords:** fructose, metabolic syndrome, metformin, vascular smooth muscle cells, osteogenic transdifferentiation, advanced glycation end-products, receptor for advanced glycation end-products

## Abstract

Metabolic syndrome (MetS) is associated with osteogenic transdifferentiation of vascular smooth muscle cells (VSMCs) and accumulation of arterial calcifications (ACs). Metformin (MET) inhibits this transdifferentiation *in vitro*. Here, we evaluate the *in vivo* efficacy of oral MET to reduce AC in a model of MetS. Twenty young male Wistar rats were divided into two groups: one received water and the other received water plus 20% fructose to induce MetS. After 14 days, and for another 4 weeks, MET (100 mg/kg per day) was added to half of each group’s drinking source, thus C (water), F (fructose), M (MET) and FM (fructose + MET). Serum and adipose tissue were collected. Aortas were dissected for histomorphometric and immunohistochemical analysis, *ex vivo* calcification studies and isolation of VSMCs to measure their alkaline phosphatase activity (ALP), collagen production, extracellular mineralization, gene expression of RUNX2 and receptor for advanced glycation end-products (AGEs) (RAGE), and elastic fiber production. F group showed parameters compatible with MetS. Aortic tunica media from F showed decreased elastic-to-muscular layer ratio, increased collagen content and increased levels of the AGEs structure carboxymethyl-lysine. Aortic arches from F presented a tendency for higher *ex vivo* calcification. VSMCs from F showed increased ALP, collagen secretion, mineralization and expression of RUNX2 and RAGE, and decreased elastic fiber production. All these effects were reverted by MET cotreatment (FM group). *In vitro*, AGEs-modified bovine serum albumin upregulated RAGE expression of control VSMCs, and this was prevented by MET in an AMP kinase-dependent manner. Thus, experimental MetS induces RAGE upregulation and osteogenic transdifferentiation of aortic VSMCs curbed by oral treatment with MET.

## Introduction

In westernized societies, the arterial system constitutes the second most mineralized site in the body after the skeleton and is one of the main sites of ectopic mineralization (1). As a consequence of pathological alterations, such as diabetes mellitus, metabolic syndrome (MetS) or renal insufficiency, or simply by physiological aging, the arterial wall acquires an important load of extracellular calcium deposits in the form of hydroxyapatite, whitlockite or octacalcium phosphate (2). Accumulation of arterial calcifications (ACs) can have severe consequences: alteration of arterial compliance that impairs distal perfusion and cardiac output, increasing the risk of life-threatening conditions, such as myocardial infarction, stroke, limb amputation, renal failure, dementia, blood vessel stenosis and ischemia (3).

In the past, accumulation of ACs was considered to be a passive process, but recent evidence shows that it is a cell-controlled and highly regulated process that involves the participation of several cell types and multiple soluble factors (4). One of the key events that lead to the accumulation of ACs is osteogenic transdifferentiation of the vascular smooth muscle cells (VSMCs) present in the tunica media of the arterial wall, caused by a plethora of pathological processes, including MetS (5, 6, 7).

Several clinical studies have shown a clear association between AC accumulation and the development of MetS or its individual components, such as obesity, hypertension, dyslipidemia, insulin-resistance and hyperglycemia (5, 8, 9). Serum of individuals with MetS and AC shows increased levels of factors that promote extracellular biomineralization: elevated levels of extracellular phosphate (10), bone-specific alkaline phosphatase (ALP) (11), osteoprotegerin (12), sclerostin (13) and osteocalcin (14). Chronic hyperglycemia induces an increase in advanced glycation end-products (AGEs) on different proteins of the extracellular matrix, and in the arterial wall, binding of accumulated AGEs to its receptor RAGE expressed by cell types such as VSMCs constitutes a crucial event for the development of AC (15, 16).

A recent preclinical study demonstrated that a partial deficit of Klotho gene in mice induces osteogenic transdifferentiation of aortic VMSCs, arteriosclerosis and hypertension, events that could be completely prevented by treatment with an AMP kinase (AMPK) activator (17). Metformin (MET) is an AMPK activator drug (18) widely used in clinical practice for the treatment of patients with type 2 diabetes mellitus, MetS and/or insulin resistance. *In vitro*, MET has previously been found to inhibit the osteogenic transdifferentiation of VSMCs (19, 20). *In vivo*, oral MET treatment can prevent the accumulation of ACs in the tunica media of rats with chronic renal disease (21) and significantly reduce the accumulation of coronary ACs, both in individuals with prediabetes (22) and in patients with type 2 diabetes mellitus (23). However, the preventive effects of MET treatment on the *in vivo* accumulation of ACs in experimental models of MetS have not been previously evaluated.

Elastic arteries, such as the aorta, are unique in that they possess circumferential elastic lamella composed of elastic fibers that alternate with VSMCs throughout the tunica media. At a whole-organ level, this facilitates the Windkessel effect, storing a portion of blood during systole and sending blood out during diastole. At a cellular level, elastic fibers and VSMCs are in close contact with each other through filaments formed by elastin extensions, which allow transmission of mechanical forces to and from VSMCs (outside-in from changes in blood pressure and inside-out due to actomyosin contraction). Elastic fiber formation is a complex process that normally occurs from mid-embryogenesis to early postnatal life, and these fibers must last the entire lifespan. Since they do not regenerate spontaneously, if they are degraded by aging and/or other conditions, this can compromise the biomechanical properties of the aortic wall (24).

The aim of the present study was to evaluate the efficacy of oral MET in preventing AC and elastin loss in an experimental model of MetS. To this effect, we have investigated the effects of fructose-induced MetS in rats (25, 26) on aortic medial histomorphometry and accumulation of AGEs, *ex vivo* calcification potential and VSMC osteogenic transdifferentiation; the *in vivo*, *ex vivo* and *in vitro* regulation of these effects by cotreatment with MET; and possible mechanisms of action involved.

## Materials and methods

### Animal treatments and experimental design

Healthy young adult Wistar male rats 12 weeks of age were housed in duos or trios in 1695 in^2^ cages with wood-shaving bedding and maintained in a temperature-controlled area at 23°C with 12 h light:12 h darkness cycle and fed standard rat laboratory chow. Initially, 20 animals were randomly divided into two groups of 10 animals each: one group received water as a drinking source, and the other received a 20% w/v fructose solution to induce MetS (Biopack, Argentina), both *ad libitum*. After 14 days, and for an additional 4 weeks, 100 mg/kg body weight per day of MET (Química Montpellier, Argentina) were added to half of each group’s drinking source (16). Thus, four groups (*n* = 5 each) were defined: C (only water), F (20% fructose solution), M (water + MET 100 mg/kg per day) and FM (20% fructose solution + MET 100 mg/kg per day). No significant differences were found in the daily water intake between all experimental groups (water alone, ±MET and ±fructose). MET dosage in drinking water was adjusted to average water intake, in order to achieve 100 mg/kg per day for each treated animal. All animal experiments were performed in conformity with the Guide on Handling and Training of Laboratory Animals published by the Universities Federation for Animal Welfare (27) and the approval for animal studies was obtained from the Institutional Animal Care and Use Committee, Faculty of Exact Sciences, National University of La Plata (CICUAL protocol 001-00-15). Group allocation was randomly assigned during the procedure. No animals were excluded during the process, and all completed the experiments uneventfully.

The rats were neither water-deprived nor fasted prior to anesthesia and euthanasia (nor were treatments administered via drinking water suspended). Before sacrifice, rats were anesthetized with xylazine 5 mg/kg, intramuscular, and ketamine 80 mg/kg, intraperitoneal (Richmond, Argentina), and then weighed, and serum samples were obtained to determine glucose, triglycerides (TG), HDL-cholesterol (HDLc), creatinine and serum activities of ALP and aspartate aminotransferase (AST) with a CM250 Metrolab Clinical Chemistry automatic analyzer using commercial kits (Wiener Lab. Group, Argentina). The TG/HDLc ratio was determined as a surrogate indicator of insulin resistance and cardiovascular risk (28). In addition, serum fructosamine was determined using a commercial kit (BioSystems, Spain), as a marker of extracellular protein glycation. Animals were euthanized by cervical dislocation under anesthesia, after which the heart, aorta and adipose tissues (mesenteric and epididymal) were dissected.

### Histomorphometric evaluation of aortic wall

Portions of the abdominal aorta were fixed in 4% v/v formaldehyde in phosphate-buffered saline (PBS) for at least 24 h and then embedded in paraffin. Four-micrometer sections were obtained with an RMT-20 Type Erma Microtome (TechLabs, India). The sections were stained with hematoxylin and eosin (H&E) or Sirius Red. Photographs were taken with a Nikon Coolpix 4500 digital camera on a Nikon ECLIPSE Ei microscope (Nikon, Japan). The images were analyzed using the ImageJ program (https://imagej.net/ij/) with a microscope scale and color deconvolution plugins. Tunica media thickness (calculated as the average distance between interior and exterior elastic membranes) and elastic-to-muscular layer ratio (calculated as the ratio of average elastic membrane width to the average thickness of muscular layer) were measured from H&E-stained sections, while the percentage of the area covered by collagen fibers was measured from Sirius Red-stained sections (29).

### Immunohistochemical evaluation of AGEs in aortic wall

Accumulation of AGEs in the aortic wall was assessed by chromogenic immunohistochemistry against the glycoxidation of AGEs structure carboxymethyl-lysine (CML). Briefly, 4 μm sections of abdominal aorta were deparaffinized and rehydrated in alcohol gradient, blocking the endogenous peroxidase activity with 0.5% H_2_O_2_. Antigen retrieval was performed with Trilogy reagent (Cell Marque, USA) according to the manufacturer’s instructions. Sections were treated with a blocking solution, incubated with mouse anti-CML antibody (1:500; Abcam, UK) for 48 h at 4°C, washed and incubated with biotinylated horse anti-mouse antibody (1:1000; Vector Laboratories, USA) for 1 h at room temperature. After another wash, sections were incubated with reagents of Vectastain Elite ABC kit (Vector Laboratories, USA) for 1 additional hour. Finally, sections were stained with a 3,3′-diaminobenzidine solution, washed and mounted. Photographs were acquired with a Nikon Coolpix 4500 digital camera on a Nikon ECLIPSE Ei microscope (Nikon, Japan) under the same light conditions in comparable areas.

### *Ex vivo* aorta calcification assay

Aortic arches were separated, placed in a culture plate and incubated in an osteogenic medium consisting of Dulbecco’s modified essential medium (DMEM) (Invitrogen, Argentina) supplemented with penicillin (100 UI/mL), streptomycin (100 μg/mL) and 10% v/v fetal bovine serum (FBS) (Natocor, Argentina) with the addition of ascorbic acid (25 μg/mL) and sodium *b*-glycerol phosphate (5 mM) for 7 days. In the case of groups C and F, this incubation was performed with or without MET 500 μM in the osteogenic medium. As the osteogenic media did not contain fructose, exposure to fructose (in F and FM groups) was always *in vivo* and for 6 weeks. Calcium content was evaluated with a method described by Holmar and coworkers, with modifications (30). Briefly, aortic arches were drained of medium, weighed and placed in HNO_3_ 0.1 N for 24 h to extract total calcium, which was then quantitated with a colorimetric kit (Wiener Lab. Group, Argentina). Individual aortic arches were weighed, and so the calcium content of each sample was normalized to sample weight (shown in Table 3 as μg of calcium per mg of aortic arch).

### Isolation and incubation of VSMCs

VSMCs were isolated from thoracoabdominal aorta rings as previously described (31). Briefly, under sterile conditions, the tunica adventitia was separated, and part of the remaining aorta was cut into 1 mm rings. Individual rings were placed in culture flasks and incubated with DMEM–20% v/v FBS and antibiotics at 37°C in a humidified atmosphere with 5% CO_2_ and 95% air. After 24 h, the medium was changed and replaced by DMEM–10% FBS and antibiotics, changing it every 2–3 days. After two weeks, when cells reached confluence, they were harvested by addition of 0.05% w/v trypsin and subcultured in multiwell plates for the different experiments described below. VSMCs were characterized by the expression of *a*-actin by immunofluorescence.

### Evaluation of VSMC osteogenic potential

VSMCs were plated at a density of 1.5 ×10^5^ cells/well in 24-well plates containing DMEM–10% v/v FBS plus antibiotics and incubated at 37°C in a humidified atmosphere with 5% CO_2_ and 95% air. When confluence was reached (after 5 days), osteogenic potential was evaluated by measuring ALP, type 1 collagen production and extracellular matrix mineralization, as described below.

For ALP measurement, cell monolayers were washed with PBS and solubilized in 500 μL 0.1% v/v Triton X-100. Aliquots of this total cell extract were used for protein determination by Bradford’s method and for spectrophotometric evaluation at 405 nm of the initial hydrolysis rate of *p*-nitrophenyl phosphate into *p*-nitrophenol at 37°C.

For evaluation of type 1 collagen production, cells were fixed with Bouin’s solution and stained with Sirius Red dye for 1 h. The stained material was dissolved with 1 mL 0.1 N sodium hydroxide, and the absorbance of the solution was measured at 550 nm (32). Extracellular calcium was evaluated with alizarin red S staining. The dye bound by mineral deposits was dissolved with 1 mL 0.1 N sodium hydroxide and quantitated spectrophotometrically at 540 nm (33). In replicate wells, cells were extracted with 500 μL 0.1% v/v Triton X-100 for protein determination by Bradford’s method.

### Evaluation of elastic fiber production by VSMCs

VSMC elastic fiber production was assessed by Weigert’s resorcin–fuchsin staining method (34) with modifications. Briefly, VSMCs were cultured in 24-well plates with DMEM–10% v/v FBS plus antibiotics for 25 days, washed with PBS and stained with Weigert’s resorcin–fuchsin reagent for 1 h. Excess dye was discarded, and the plate was washed with 96% v/v ethanol. The dye attached to elastic fibers was extracted with 500 μL dimethyl sulfoxide for 24 h and quantitated spectrophotometrically at 565 nm. In replicate wells, cells were extracted with 500 μL 0.1% v/v Triton X-100 for protein determination by Bradford’s method.

### Evaluation of VSMC gene expression

VSMC gene expression of runt-related transcription factor 2 (RUNX2, key marker of osteogenic transdifferentiation) and RAGE (receptor implicated in the osteogenic switch of VSMCs) was evaluated using reverse transcription-polymerase chain reaction (RT-PCR). Cells were seeded in six-well plates and cultured in DMEM–10% v/v FBS until confluence was reached. Total RNA was then extracted using the QUICK-ZOL reagent according to the manufacturer’s instructions (Kalium Technologies, Argentina). Retrotranscription was performed using Moloney murine leukemia virus reverse transcriptase (Productos Bio-Lógicos, Argentina). Specific primers were designed from the NCBI sequence database using the CLC Genomics Workbench software QIAGEN and manufactured by Macrogen (Republic of Korea) (Table 1). After the RT-PCR products were separated by agarose gel electrophoresis and visualized with GelRed, the intensity of the bands was quantitated with ImageJ using *b-actin* as a housekeeping gene.

**Table 1:** Primer sequences for RT-PCR.

Marker	Genbank code	Size (bp)	Sequence	
β-actin	NM_031144.3	345	Fw	CCT​TCA​ACA​CCC​CAG​CCA​T
			Rv	CAT​AGC​TCT​TCT​CCA​GGG​A
Runx2 (2 bands)	NM_001278483.2	598/424	Fw	GCC​GGG​AAT​GAT​GAG​AAC​TA
			Rv	TGAGAGAGGAAGGCCAGA
RAGE	NM_053336.2	1109	Fv	GTCAGAACATCACAGCC
			Rv	CGCTTCCTCTGACTGATT

### *In vitro* effect of MET and AGEs on RAGE gene expression of control VSMCs

AGEs-modified bovine serum albumin (AGEs-BSA) was obtained by incubating 10 mg/mL cell culture-grade BSA (Sigma-Aldrich, USA) under sterile conditions, with 33 mM D-glycolaldehyde in 150 mM PBS of pH 7.4 at 37°C for 3 days. Excess sugar was removed by centrifugation/filtration. Formation of AGEs was confirmed and quantitated by fluorescence emission at 420 nm upon excitation at 340 nm. Non-glycated (control) BSA was obtained by excluding D-glycolaldehyde from the incubation mixture. Control VSMCs (from C group) were cultured in six-well plates until confluence. The medium was changed, and cells were incubated for 72 h in DMEM–5% v/v FBS with 100 μg/mL AGEs-BSA or non-glycated BSA in the presence or absence of 500 μM MET and/or 0.5 μM compound C (dorsomorphin, a potent inhibitor of AMPK activation/phosphorylation). After the 72-h incubation, the gene expression of RAGE was assessed by RT-PCR and agarose gel electrophoresis as described above.

### Statistical analysis

Animal sample size was calculated by power analysis based on previous experiments (16). Results are expressed as mean ± standard error of the mean. Assumptions of normal distribution and homoscedasticity were checked by the Shapiro–Wilk and Bartlett tests, respectively. One-way ANOVA with Tukey’s post hoc test was performed using the GraphPad software (San Diego, USA); *P* values < 0.05 were considered statistically significant.

## Results

### General observations and serum profile

No significant differences were found in body weight and relative weight of cardiac and epididymal adipose tissues between groups at the end of the study. We did not evaluate blood pressure or perform an oral glucose tolerance test (both are components of MetS) and did not assess systemic or local inflammation levels (as possible contributors to cardiovascular stress), constituting a limitation of our study. In any case, animals from F group showed several other changes compatible with MetS: a significant increase in mesenteric adiposity, in non-fasting glycemia and in TG and TG/HDLc ratio (compared to C group). In particular, animals exposed to fructose alone showed significantly higher non-fasting glycemia than those in C and M groups; this effect was completely normalized by cotreatment with MET (Table 2). Creatinine, ALP and AST were also measured to evaluate possible renal damage and/or hepatotoxicity; however, no significant differences were observed for these parameters among experimental groups (data not shown).

**Table 2:** Anatomical parameters and non-fasting serum profiles after six weeks of fructose and/or four week metformin treatments.

	C	F	M	FM
Body weight (g)	284 ± 22	268 ± 39	278 ± 26	261 ± 48
Mesenteric adipose tissue (% of bodyweight)	1.36 ± 0.26	1.94 ± 0.23^#^	1.53 ± 0.19	1.62 ± 0.28
Epididymal adipose tissue (% of bodyweight)	1.94 ± 0.48	2.19 ± 0.30	2.04 ± 0.26	1.96 ± 0.28
Cardiac tissue (% of bodyweight)	0.35 ± 0.07	0.30 ± 0.08	0.31 ± 0.01	0.32 ± 0.06
Glucose (g/dL)	1.78 ± 0.09	3.22 ± 0.19^***^	2.12 ± 0.07	2.18 ± 0.14
Triglycerides (mg/dL)	48.0 ± 5.6	121.8 ± 22.6^#^	87.8 ± 18.4	97.2 ± 12.8
TG/HDLc ratio	2.5 ± 0.2	5.6 ± 1.1^#^	2.82 ± 0.8	4.7 ± 0.7

C: control group; F: rats receiving 20% fructose solution; M: rats receiving 100 mg/kg per day metformin; FM: rats receiving fructose plus metformin. Differences:

^#^ *P* < 0.05 vs C;

*** *P* < 0.001 vs C, M and FM. Results are expressed as mean ± SEM.

### *In vivo* treatment with MET reverts MetS-induced histological alterations of the arterial wall

**Figure 1:**
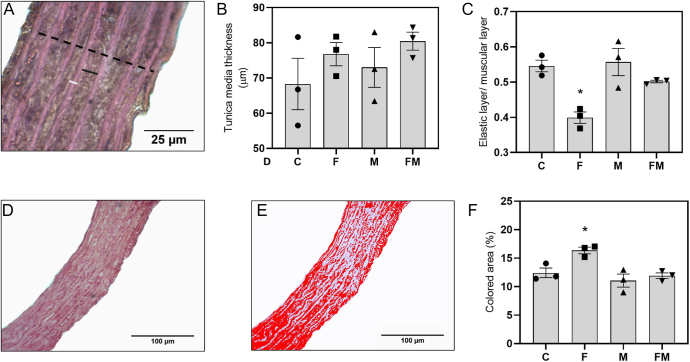
Histomorphometric analysis of aortic sections. (A) The black dotted line shows the distance between external and internal elastic membranes, and the black and white lines represent the width of individual muscular and elastic layers, respectively (H&E), obj. ×100. (B) Comparison of average tunica media thickness. (C) Comparison of average elastic-to-muscular layer ratio. (D) Representative micrography of aortic section stained with Sirius Red, obj. ×40. (E) The red area represents the area covered by collagen, obj. ×40. (F) Quantitative analysis of area covered by collagen. C: control group; F: rats receiving 20% fructose solution; M: rats receiving 100 mg/kg per day metformin; FM: rats receiving fructose plus metformin. Differences: *P < 0.05 vs C, M and FM. Results are expressed as mean ± SEM. *n* = 3 per experimental group.

Although no significant differences were found for overall tunica media thickness among groups (Fig. 1B), animals exposed only to fructose showed a significant decrease in tunica media elastic-to-muscular layer ratio (Fig. 1C). This deleterious effect was completely abrogated by *in vivo* cotreatment with MET (Fig. 1C).

Aortic sections of the F group stained with Sirius Red showed a significant increase in arterial wall collagen content, compared to the other experimental groups. Oral cotreatment with MET (FM) completely reverted this effect (Fig. 1F).

### MET can reduce the calcification of aortas from animals with MetS

Aortic arches of animals exposed only to fructose (group F) exhibited a higher predisposition to *ex vivo* calcification when incubated for 7 days in an osteogenic medium, showing a 60% increase in calcium accumulation (compared to aortic arches from control animals). MET, either orally administered *in vivo* (FM group) or added *in vitro* to the osteogenic medium for aortic arches from animals of F group (F + MET), tended to decrease fructose-induced calcium accumulation of the arterial wall (Table 3).

**Table 3:** Calcium content of aortic arches cultured in osteogenic medium.

Condition	Calcium content (μg Ca^+2^/mg of tissue)
C	7.76
C + MET	5.40
F	12.54
F + MET	5.19
M	5.12
FM	4.43

C: control group; C+MET: aortic arches of C with 500 μM metformin in osteogenic medium; F: rats receiving 20% fructose solution; F+MET: aortic arches of F with 500 μM metformin in osteogenic medium; M: rats receiving oral metformin; FM: rats receiving oral fructose plus metformin.

### Effect of MetS and oral MET on VSMC osteogenic potential

**Figure 2:**
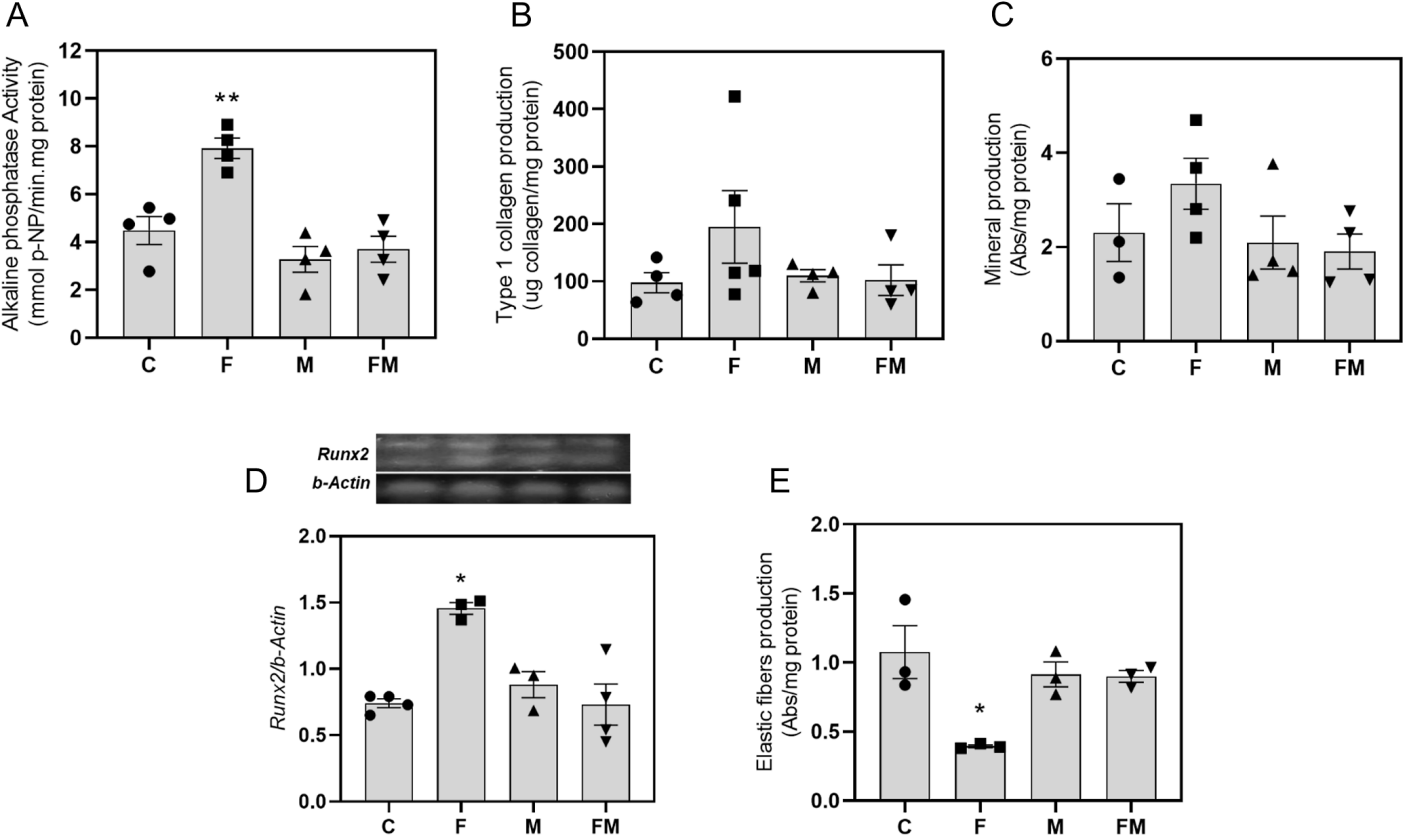
Effects of fructose and/or metformin treatments on different markers of VSMC osteogenic potential: (A) alkaline phosphatase activity, (B) type 1 collagen production, (C) mineralization of extracellular matrix, (D) relative gene expression of RUNX2 by RT-PCR and (E) quantification of elastic fibers. C: control group; F: rats receiving 20% fructose solution; M: rats receiving 100 mg/kg per day metformin; FM: rats receiving fructose plus metformin. Differences: *P < 0.05 vs C, M and FM; **P < 0.01 vs C, M and FM. Results are expressed as mean ± SEM. *n* = 3–5 per experimental group.

VSMCs isolated from animals of the F group presented a significant increase in ALP (Fig. 2A), a tendency for higher type 1 collagen production (Fig. 2B) and for mineralization of the extracellular matrix (Fig. 2C), and a significant increase in the expression of osteogenic marker *Runx2* (Fig. 2D). Cotreatment with oral fructose and MET (FM group) completely reverted these effects.

### Effect of MetS and oral MET on VSMC elastic fiber production

VSMCs were cultured for 25 days and then evaluated for elastic fiber production. A significant decrease was observed in cells isolated from animals of group F (versus group C). Cotreatment with fructose and MET (group FM) completely reverted this effect (Fig. 2E).

### Effect of MetS and oral MET on extracellular protein glycation and on VSMC gene expression of receptor for advanced glycation end-products

**Figure 3:**
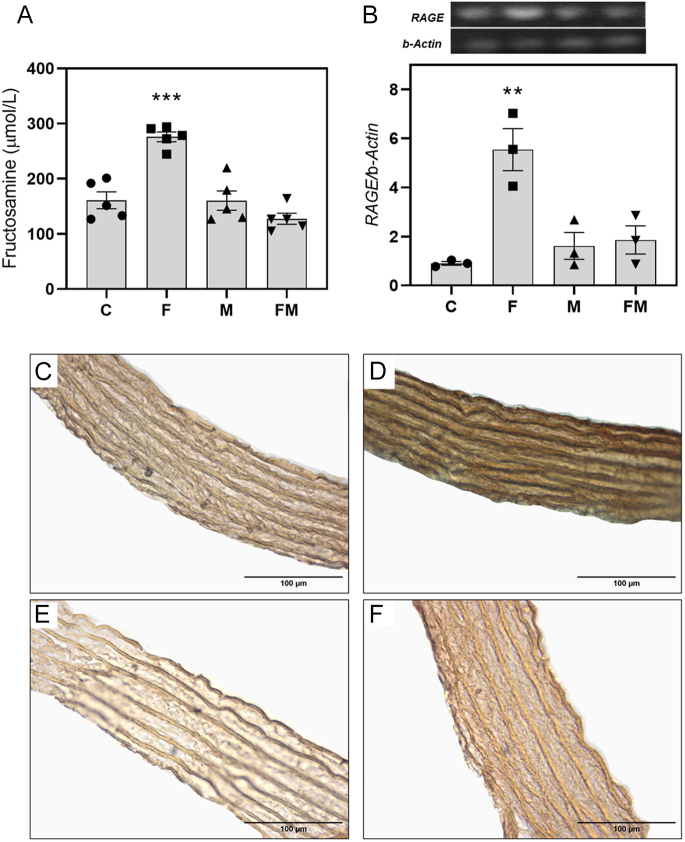
Effect of fructose and/or metformin treatment on (A) serum fructosamine levels, (B) VSMC relative gene expression of RAGE by RT-PCR. Carboxymethyl-lysine extracellular accumulation in the aortic wall (by immunohistochemistry, obj. ×40) of (C) control rats (C group), (D) rats receiving 20% fructose solution (F group), (E) rats receiving 100 mg/kg per day metformin (M group) and (F) rats receiving fructose plus metformin (FM group). Immunohistochemical staining of CML was performed in aortic sections of all animals, and representative photographs are shown in panels (C–F). Differences: **P < 0.01 vs C, M and FM; ***P < 0.001 vs C, M and FM. Quantitative results are expressed as mean ± SEM. *n* = 3–5 per experimental group.

Animals from group F presented significantly higher levels of serum fructosamine, a marker of systemic extracellular protein glycation (Fig. 3A). Furthermore, the same group of animals showed a significant increase in VSMC gene expression of RAGE, the main cell receptor for AGEs (Fig. 3B). In addition, immunohistochemical detection of carboxymethyl-lysine (CML, a prevalent AGEs structure) was performed in the aortic sections of animals from all experimental groups (Fig. 3C–F). CML was found to be visibly increased (versus the control) in the extracellular material from the tunica media of fructose-exposed animals (Fig. 3D). All these effects were completely reverted when fructose and MET were co-administered orally (group FM, Fig. 3F).

### *In vitro* effects of AGEs and MET on gene expression of RAGE in VSMCs: role of AMPK activation

**Figure 4:**
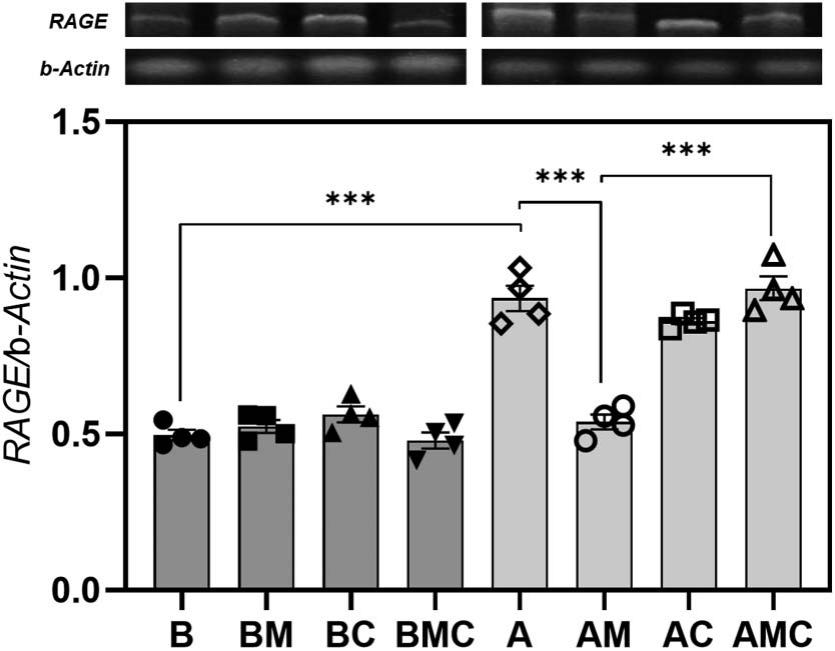
*In vitro* effects of metformin and/or the AMPK inhibitor compound C on RAGE relative gene expression of control VSMCs (from C group). B: 100 μg/mL non-glycated bovine serum albumin (BSA); BM: 100 μg/mL BSA plus 500 μM metformin; BC: 100 μg/mL BSA + 0.5 μM compound C; BMC: 100 μg/mL BSA +500 μM metformin + 0.5 μM compound C; A: 100 μg/mL AGEs–BSA; AM: 100 μg/mL AGEs–BSA + 500 μM metformin; AC: 100 μg/mL AGEs–BSA +0.5 μM compound C; AMC: 100 μg/mL AGEs–BSA + 500 μM metformin + 0.5 μM compound C. Differences: ***P < 0.001. Results are expressed as mean ± SEM. *n* = 4 per experimental group.

We performed *in vitro* experiments, in which we cultured VSMCs obtained from control animals for 72 h with AGEs-BSA and/or MET and/or compound C (a potent inhibitor of AMPK activation/phosphorylation). AGEs-BSA significantly upregulated the VSMC relative gene expression of RAGE (versus non-glycated BSA). This upregulation was completely prevented when VSMCs were co-incubated with AGEs-BSA and MET. Prevention by MET of AGEs-dependent RAGE upregulation was abrogated by the AMPK inhibitor compound C (Fig. 4).

## Discussion

Although accumulation of vascular calcification and associated cardiovascular disease (CVD), such as myocardial infarction, stroke, blood vessel stenosis and ischemia, are usually associated with modern society and current lifestyles, in reality, they constitute pathological entities that have accompanied us since the birth of civilization. This has been shown by the Horus study, in which ectopic biomineralization was demonstrated in the aorta, carotid, coronary, femoral, iliac and peripheral leg arteries of mummified individuals of ancient Egypt who lived over 3500 years ago (35).

The current rise in sedentarism, obesity and high fructose intake (due to industrial use of high-fructose corn syrup in beverages) strongly correlates with an increased incidence of metabolic disorders, such as MetS and type 2 diabetes mellitus, causing, at the same time, a higher prevalence of AC, making the population more prone to suffer from CVD, which has constituted the leading cause of death worldwide for the past decades (36).

Due to its high effectiveness and safety supported by more than 60 years of clinical experience, and its relatively low cost, MET is included in the first-line pharmacological therapy for alterations of glucose metabolism.

Since the UKPDS in 1998 evidenced how intensive glucose control and the early use of MET could reduce cardiovascular complications and mortality up to 40% in overweight patients suffering from type 2 diabetes mellitus (37), several other clinical studies have shown the clear efficacy of MET in reducing the burden of AC in patients with MetS and/or diabetes. For example, in the DIACART study, patients with diabetes treated with MET showed a significantly lower AC score in below-the-knee arteries than those who did not use that drug (38), while in a randomized placebo-controlled study performed on patients with MetS positive for HIV, MET successfully limited plaque progression and reduced the AC score compared to placebo and even compared to lifestyle modifications (39). In line with these findings, a 14-year follow-up study found lower AC scores in patients with prediabetes when they were treated with MET (40). Thus, MET has been shown in clinical trials to be strongly associated with the lowering of AC, suggesting a promising additional effect of this antidiabetic drug (38). It has also been shown that MET is able to reduce other changes in the vessel wall, resulting in a decrease in carotid intima-media thickness in MET-treated patients with diabetes (41).

In the present study, we used 20% fructose in drinking water to induce MetS. Given that our goal was to observe cardiovascular outcomes, a combined high-fructose/high-fat model for MetS may have been more appropriate and could be a limitation of this study. In any case, our preclinical model of a high-fructose diet alone induced physiological and metabolic abnormalities similar to those of human MetS, such as hyperglycemia, hypertriglyceridemia, high TG/HDLc ratio and increased mesenteric adiposity, while cotreatment of fructose with MET tended to mitigate those alterations. These findings support the validity of our fructose-treated group as a model for MetS.

We also found in this study that animals exposed to a high-fructose diet for six weeks developed histological alterations of the aortic wall, demonstrated by a reduction in the elastic-to-muscular layer ratio, an increase in collagen content and in the accumulation of extracellular AGEs and a tendency toward an increased potential for calcium accumulation in the aortic arch. Importantly, all of these fructose-induced aortic histological changes could be completely reverted by cotreatment with MET. Several vascular changes have been shown to contribute to arterial tunica media stiffness, which include fibrosis or excessive deposits of collagen fibers, accumulation of ACs associated with collagen and elastin fibers, fragmentation of elastic fibers (elastinolysis) and irreversible cross-linking of collagen and/or elastic fibers due to accumulation of AGEs (1).

The vascular extracellular matrix remodeling favors the accumulation of ACs by VSMCs, which in turn causes stiffening of vessel walls. This process is accelerated by increased expression of matrix proteases, such as calpain-1 and matrix metalloproteinase-2 (MMP2), that are upregulated in aged human aortic walls and atherosclerotic lesions. Studies with VSMCs *in vitro* and with carotid artery rings *ex vivo*, have shown that increased activity of calpain-1 and MMP2 promotes VSMC-induced calcifications, ALP-activation, elastin degradation and collagen accumulation (42).

In agreement with our histological observations, in this study, we found that VSMCs isolated from the aortas of fructose-treated rats showed increased levels of *Runx2* expression and ALP activity (versus VSMCs from control animals) and a tendency toward higher type 1 collagen secretion and mineral nodule production, while all fructose-induced effects on VSMCs were completely reverted by oral cotreatment with MET.

Other authors have previously reported that the *in vitro* addition of MET to rat aortic VSMCs cultured in an osteogenic medium attenuates their predisposition to acquire an osteoblastic phenotype, potentially preventing accumulation of vascular calcifications, via an AMPK–eNOS–NO pathway (19). Interestingly, in the present study, we found increased markers of osteoblastic phenotype in VSMCs freshly isolated from fructose-treated animals and cultured in basal medium (without the use of external osteogenic inducers, such as *b*-glycerol phosphate and/or high concentrations of calcium), effects that were completely reverted by oral coadministration of MET. This may be due to a direct regulation of osteogenic genes in VSMCs by fructose and/or MET and/or could also be the result of epigenetic regulation of gene expression, as has been previously shown by other authors (43).

In the present study, in addition to the fructose-induced osteogenic transdifferentiation of VSMCs, we also observed a significant decrease in their *ex vivo* capacity to produce elastic fibers, an effect that was reverted in VSMCs isolated from rats that had been simultaneously treated with fructose and MET. These *ex vivo* results correlate with our *in vivo* aortic elastic-to-muscular layer ratio observations. All in all, our results indicate that experimental MetS can induce structural remodeling of the aortic wall, leading to modifications in the balance between elastic and collagen fibers, changes that may increase vessel stiffness and loss of arterial compliance, affecting its physiological functions (44). Further research is needed to determine whether these effects are caused by a reduced capacity to produce elastic fibers and/or by an increased rate of elastinolysis.

A considerable number of *in vivo, ex vivo* and *in vitro* preclinical studies have attempted to elucidate and explain possible molecular mechanisms involved in the protective effects shown by MET on VSMC osteoblastic transdifferentiation. Phadwal and coworkers found that *in vitro* MET promotes the autophagic degradation of RUNX2, thus preventing calcification in a dose-dependent manner (20), while in another study, pre-treatment with *in vivo* or *in vitro* MET was able to mitigate hyperlipidemia-associated accumulation of vascular calcifications by enhancing ferroptosis and to enhance the antioxidative capacity of VSMCs via p53 and Nrf2 signaling (45). *In vitro* MET was also shown to inhibit phosphate-induced VSMC osteogenic transdifferentiation via AMPK activation and subsequent inhibition of RANKL (46).

Previous work from our laboratory has demonstrated that VSMCs cultured *in vitro* in the presence of AGEs exhibit an increase in the markers of osteoblastic phenotype (increased RUNX2 expression, ALP activity, type 1 collagen production and extracellular mineralization), losing at the same time the markers of smooth muscle phenotype (16, 32). In the present study, serum from fructose-treated rats showed an increase in fructosamine, a marker of extracellular protein glycation, and potentially of an increase in systemic AGEs. In addition, by immunohistochemical staining, we found an increase in the extracellular CML (a prevalent AGEs structure) in the aortic tunica media of fructose-treated rats. Both serum fructosamine and aortic CML were reverted to control levels in rats cotreated with fructose and MET.

AGEs-induced VSMC osteogenic transdifferentiation is believed to be caused by the interaction of AGEs with their RAGE, expressed in this cell type (1). AGEs–RAGE binding activates the Diaphanous 1 pathway and generates intracellular oxidative stress, inducing the translocation of NF-kB transcription factor via Ras and MAPK activation, increasing the expression of multiple genes, including pro-osteogenic genes and of RAGE itself (47, 48). This upregulation of RAGE can be expected to further amplify the cellular consequences of AGEs accumulation. In the present study, in animals treated only with fructose, we observed a simultaneous increase in aortic VSMC gene expression of RAGE and in extracellular AGEs accumulation in the aortic tunica media. Importantly, both effects were completely reverted when fructose was coadministered with MET. In other experiments, we also cultured VSMCs from control animals with either AGEs-BSA or non-glycated BSA in the culture media (in the presence or absence of MET and/or compound C, which is an inhibitor of AMPK activation/phosphorylation). In these *in vitro* experiments, we found that while AGEs-BSA (versus non-glycated BSA) upregulated RAGE expression of VSMCs, this upregulation was completely prevented by coincubation with MET (apparently by an AMPK-dependent mechanism, since prevention was abrogated by compound C).

In previous studies with VSMCs from rats with diabetes, we have found that *in vitro* and *in vivo* increased levels of AGEs can induce their osteoblastic transdifferentiation and that these effects are completely prevented by *in vitro* or *in vivo* MET, respectively (16). In other *in vivo* studies with bone marrow stromal cells from diabetic rats, we have shown that an increase in extracellular protein glycation is associated with RAGE upregulation and that this effect can be completely prevented by oral treatment with MET (49).

Great caution must be taken when attempting to extrapolate our present findings to humans; nevertheless, the results of previously published clinical trials indicate that oral MET could contribute to reduced AC in individuals with MetS (39), prediabetes (22, 40) and type 2 diabetes mellitus (23, 38). Further studies involving quantitation of protein expression, evaluation of inflammation and/or oxidative stress markers are necessary to fully elucidate the proposed mechanism of action based on AGEs–RAGE interaction and the role of MET in reverting the observed pathological alterations induced by a fructose-rich diet. Future evaluation of histones and DNA modifications must be performed to evaluate the epigenetic markers that could be related to the pro-osteogenic cellular imprint found in freshly isolated VSMCs.

In conclusion, in our present study, we have found that a fructose-rich diet in rats induces metabolic alterations compatible with the development of MetS, additionally causing increased serum protein glycation; in the aortic tunica media, decreased elastic-to-muscular layer ratio, increased collagen content, increased AGEs accumulation and procalcifying potential; and in VSMCs isolated from aortas, induction of an osteoblastic phenotype, decreased elastic fiber production and increased RAGE expression. All of these MetS-induced aortic structural and cellular phenotypic changes (which may be partly due to increased activation of upregulated RAGE in aortic VSMCs) can be completely reverted by oral cotreatment with MET, probably via AMPK activation.

## Declaration of interest

The authors declare that there is no conflict of interest that could be perceived as prejudicing the impartiality of the work reported. Dr Antonio McCarthy is a Senior Editor of *Endocrine Connections*. Dr McCarthy was not involved in the review or editorial process for this paper, in which he is listed as an author.

## Funding

This work was supported by Universidad Nacional de La Plata (UNLP)https://doi.org/10.13039/501100003947 (Proyecto de incentivos, Proyectos 11/X768-X945), Comisión de Investigaciones Científicas de la Provincia de Buenos Aires (CICPBA) (PIO-CICPBA-CONICET-2015/2016-20920150100002CO) and Agencia Nacional de Promoción Científica y Tecnológica (PICT-Raíces-2015-1361; PICT-2015-1030; PICT-2021-CAT-I-00114).

## Author contribution statement

Lucas Streckwall contributed to investigation, formal analysis, writing of the original draft and visualization. Nancy Martini helped in investigation, formal analysis, writing of the original draft and visualization. Claudia Sedlinsky helped in conceptualization and reviewing and editing of the manuscript. León Schurman helped in conceptualization, resources and reviewing and editing of the manuscript. María Virginia Gangoiti contributed to conceptualization, methodology, validation, reviewing and editing of the manuscript and supervision. Antonio Desmond McCarthy helped in conceptualization, methodology, validation, funding acquisition, reviewing and editing of the manuscript, supervision and project administration.
